# Analysis of Antibody Aggregate Content at Extremely High Concentrations Using Sedimentation Velocity with a Novel Interference Optics

**DOI:** 10.1371/journal.pone.0120820

**Published:** 2015-03-24

**Authors:** Kristian Schilling, Frank Krause

**Affiliations:** Nanolytics Gesellschaft für Kolloidanalytik, Potsdam, Germany; Kermanshah University of Medical Sciences, IRAN, ISLAMIC REPUBLIC OF

## Abstract

Monoclonal antibodies represent the most important group of protein-based biopharmaceuticals. During formulation, manufacturing, or storage, antibodies may suffer post-translational modifications altering their physical and chemical properties. Such induced conformational changes may lead to the formation of aggregates, which can not only reduce their efficiency but also be immunogenic. Therefore, it is essential to monitor the amount of size variants to ensure consistency and quality of pharmaceutical antibodies. In many cases, antibodies are formulated at very high concentrations > 50 g/L, mostly along with high amounts of sugar-based excipients. As a consequence, all routine aggregation analysis methods, such as size-exclusion chromatography, cannot monitor the size distribution at those original conditions, but only after dilution and usually under completely different solvent conditions. In contrast, sedimentation velocity (SV) allows to analyze samples directly in the product formulation, both with limited sample-matrix interactions and minimal dilution. One prerequisite for the analysis of highly concentrated samples is the detection of steep concentration gradients with sufficient resolution: Commercially available ultracentrifuges are not able to resolve such steep interference profiles. With the development of our Advanced Interference Detection Array (AIDA), it has become possible to register interferograms of solutions as highly concentrated as 150 g/L. The other major difficulty encountered at high protein concentrations is the pronounced non-ideal sedimentation behavior resulting from repulsive intermolecular interactions, for which a comprehensive theoretical modelling has not yet been achieved. Here, we report the first SV analysis of highly concentrated antibodies up to 147 g/L employing the unique AIDA ultracentrifuge. By developing a consistent experimental design and data fit approach, we were able to provide a reliable estimation of the minimum content of soluble aggregates in the original formulations of two antibodies. Limitations of the procedure are discussed.

## Introduction

Therapeutic proteins such as monoclonal antibodies have been enjoying increasing significance in the biopharmaceuticals market, major therapeutic areas being cancer and immune/inflammation-related disorders [1–3]. The characterisation of monoclonal antibodies (mAbs) is a major challenge in process monitoring and quality control. The main product characteristics to be monitored are aggregate and fragment content, glycosylation pattern and charge variants [4, 5].

In many cases, antibodies are formulated at high concentrations > 50 g/L, often in buffers with high amounts of sugar-based excipients. Therefore, the size distribution at those original conditions cannot be monitored using routine aggregation analysis methods, such as size-exclusion chromatography. This is only possible after dilution (down to not more than 1–2 g/L) and usually under significantly altered solvent conditions [6–8]. In consequence of such an invasive sample preparation, these assays may not accurately determine the non-covalent higher molecular weight forms occurring in the original formulation [6, 7].

In contrast, sedimentation velocity (SV) allows to analyze samples directly in the product formulation, both with limited sample-matrix interactions and minimal dilution. For these reasons, SV is considered an accuracy standard for quantitation of protein aggregation [9].

Sedimentation velocity provides important information on macromolecules in solution. Two measurements are of particular value for characterizing biopharmaceutical protein products: the monomer sedimentation coefficient and the amount of protein aggregation. Sedimentation coefficient measurements are highly precise and can provide a sensitive probe of conformational properties and changes [9]. In this study, however, we focus specifically on the application of SV for the measurement of protein aggregation.

In biopharmaceutical industry, the c(s) distribution method implemented in the software program SEDFIT has become a popular tool for analyzing SV data [10–12], particularly for quantifying trace levels of aggregation in therapeutic proteins [9]. The c(s) distribution method provides a convenient resource for analysts to model complex SV raw data (concentration as a function of radial position and time) using numerical Lamm equation solutions to achieve a distribution of relative concentration as a function of the sedimentation coefficient [11–13].

So far, the most important advantage of SV in characterising pharmaceutical antibodies has been sacrificed to meet requirements for approximately ideal sedimentation behavior as well as to allow for absorbance detection as the standard optics used [9, 14–19]. For this purpose, the formulated antibodies were diluted to concentrations below 1–2 g/L and frequently even formulation components, such as sugar excipients, were omitted, by applying standard salt buffers.

There is a fundamental technical challenge in directly analysing highly concentrated protein solutions, requiring the SV analysis of extremely steep boundaries [20–22]. The commercially available XL-I ultracentrifuge introduced more than 20 years ago should fail in imaging gradients steeper than 375 fringes/cm (refer to Results). In practice, however, the XL-I system is observed to fail at lower gradients, e.g. when recording fringe gradients of antibodies at 100 g/L measured in cells of the lowest tractable optical pathlength of 1.5 mm (refer to Results).

As an important theoretical limitation, non-ideal sedimentation behavior is a consequence of unspecific intermolecular interactions between solute molecules. These lead to both a decrease in sedimentation velocity (i.e. the sedimentation coefficient) as well as a hypersharpening of sedimentation boundaries. However, the Lamm equation (1) does not account for intermolecular interactions. For concentrations of typical proteins above 1–2 g/L, the excluded volume contribution to non-ideality will start to become significant [13, 17].

$$\frac{\partial c}{\partial t}=\frac{1}{r}\frac{\partial }{\partial r}\left[r\cdot D\frac{\partial c}{\partial r}-s\omega^{2}r^{2}c\right]$$ (1)

In this study, we report the first results of a unique in-house-developed interference detector (Aida, Advanced Interference Detection Array), capable of recording steep fringe gradients generated by high solute concentrations. As a proof-of-concept, we analyzed two antibodies formulated at concentrations of 102 and 147 g/L, respectively.

In this work we comprehensively optimized the experimental procedure and data fit approach, minimizing the impact of both steep fringe gradients and non-ideal sedimentation behavior on the precision of the results.

The data fit approach was based on the c(s) distribution with floating frictional ratio f/f_0_ and proved to provide a reliable estimation of the minimum content of soluble aggregates despite significant non-ideal sedimentation behavior. This study presents the first size distribution analysis of an antibody at very high concentrations in complex formulations containing high amounts of redistributing sugar excipients.

## Materials and Methods

### Antibodies

The antibodies were provided by Sanofi-Aventis Deutschland GmbH (Frankfurt/Main, Germany). Antibody 1 is formulated at a concentration of 147 g/L in 10 mM citrate, pH 5.5, 40 g/L mannitol, 0.05 g/L Polysorbate 20.

Antibody 2 is formulated at a concentration of 102 g/L in 10 mM citrate, 45 g/L sucrose, 10 g/L arginine-hydrochloride, 2 g/L NaCl, 0.1 g/L Polysorbate 20.

### Setup of the Aida ultracentrifuge

**Fig 1:**
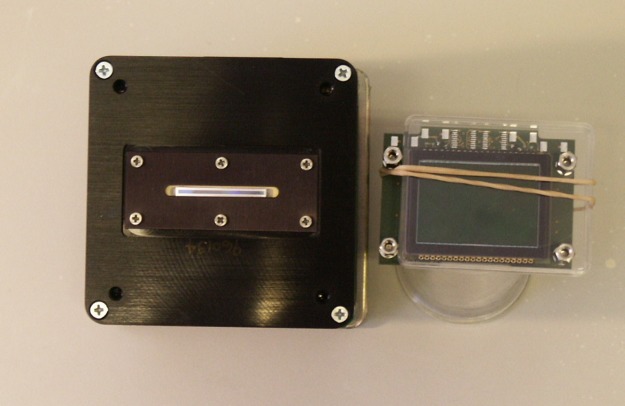
Interference detectors of the Optima XL-I ultracentrifuge (left) and the custom-built Aida ultracentrifufe (right). The larger camera surface of Aida allowed for a higher optical magnification factor of 2 vs. 1.75 in the XL-I centrifuge after adapting the lens system accordingly. Additional improvements are described in the text.

To substantially improve the resolution capability of the Rayleigh interference optics provided by the standard Analytical Ultracentrifuge—BeckmanCoulter’s Optima XL-I—we introduced a state-of-the-art CCD camera as the main element of the Advanced Interference Detection Array (Aida) as shown in Fig. 1. This best available Rayleigh interference optics was integrated into a preparative BeckmanCoulter centrifuge.

The Aida detector is capable of recording steep fringe gradients generated by highly concentrated protein solutions. Similarly, the resolution of interference data is significantly increased allowing for the analysis of diluted solutions down to a range of 0.01–0.03 g/L, at which interference data quality of the Optima XL-I is too low for an accurate quantitative analysis.

Additional improvements include modifications of the detector periscope. (1) The total optical system, including light source, lens and mirror optics, and CCD detector, is one geometrical entity, passing through the vacuum chamber bottom with a flexible feedthrough. This ensures that slight movements of the chamber bottom during activity of the Peltier elements do not propagate into misalignments of the light source vs. detection units, as observed when the light source is mounted onto the chamber bottom and the optical path feeds through at another position. (2) The cylinder lens is mounted into a three-axis assembly that permits more sensitive adjustment of this critical component than in the commercially available setup. Both modifications follow suggestions made by Thomas M. Laue, eliminating two substancial sources of error in the currently available system (Laue, TM (2006) personal communication.). Additionally, the larger camera surface allowed for a higher optical magnification factor of 2 vs. 1.75 in the XL-I centrifuge after adapting the lens system accordingly.

### Analytical Ultracentrifugation sedimentation velocity

AUC-SV analysis was performed according to the experimental protocol developed for the present study as outlined in the Results. It addresses specific requirements for measuring the aggregation content in originally formulated antibody solutions at high concentrations of 100–147 g/L. The data fit procedure was developed simultaneously.

Dilutions were freshly prepared prior to measurement using the respective formulation buffer.

Samples were filled into custom-produced titanium centerpieces with sapphire windows with pathlengths of 3 or 12 mm at concentrations up to 10 mg/mL and 1.5 mm at concentrations >10 mg/mL, respectively. Titanium as a cuvette material ensures optimal chemical resistance, minimal surface roughness, and maximum mechanical stability. Upon inserting the cells into the rotor, optical alignment along the centrifugal field is ensured by the application of our custom-made cell alignment tool. It has been shown to be essential that the walls of the cuvette, sector shaped in order to align perfectly with the centrifugal field, must not be rotated out of the ideal position by more than few 10ths of a degree. Errors in alignment have been identified as one of the most important sources of inter-run and intra-run variability for protein aggregation measurements [9].

Buffer density and viscosity were calculated incrementally using Sednterp 2.0 according to the given composition. A standard value for globular proteins with a glycosylation degree of 3% (w/w) was used for the partial specific volume (0.72 mL/g).

Sedimentation velocity experiments of antibody solutions above 10 g/L were carried out on a Beckman L-60 preparative ultracentrifuge equipped with a custom built Advanced Interference Detection Array (Aida) at 20°C and an angular velocity of either 25 krpm or 40 krpm. Diluted antibodies up to concentrations of 10 mg/mL were analyzed on a BeckmanCoulter XL-A/XL-I Analytical Ultracentrifuge using interference and absorbance optics at 20°C and an angular velocity of 40 krpm.

The data were analyzed with the standard c(s) model [10] in SEDFIT version 14.4 (https://sedfitsedphat.nibib.nih.gov/software/default.aspx).

All plots of AUC raw data, best fits and residuals were created with the software GUSSI, which can be downloaded from the MBR Software Page (http://biophysics.swmed.edu/MBR/software.html). Data plots of c(s) distributions were created by in-house developed software.

## Results

### Experimental enhancements generating high quality SV data at loading concentrations above 50 g/L

For monitoring the amount of monomers and oligomers/aggregates in antibodies, we analyzed two samples using sedimentation velocity (SV) with the Aida detector. The two antibodies were formulated at concentrations of 102 g/L and 147 g/L, respectively, in distinct citrate buffers with a similar content of sugar osmolytes.

The experimental design and data fit approach was optimized during the course of this work as outlined below.

### Use of interference window holders

The use of interference window holders is recommended for interference optics [23]. Interference window holders enhance image quality, as they block stray light from the light source, presumably diffracted at the slit exit. Thus, secondary interference events are minimized, and the image appears much sharper.

Since our Advanced Interference Detection Array (Aida) accesses a much larger region of the interferogram than the XL-I ultracentrifuge, the benefit of enhancing the quality of the image in the entire region is even more obvious. We observed a significantly improved quality of interferograms by employing interference window holders on highly concentrated antibody solutions. In contrast to diluted solutions, we also found that the improved image quality results in better quality of the Fourier transformed scans: they exhibit less noise and improved stability in the region of steep fringes when analyzing highly concentrated solutions.

### Precise camera alignment and Fourier transform

**Fig 2:**
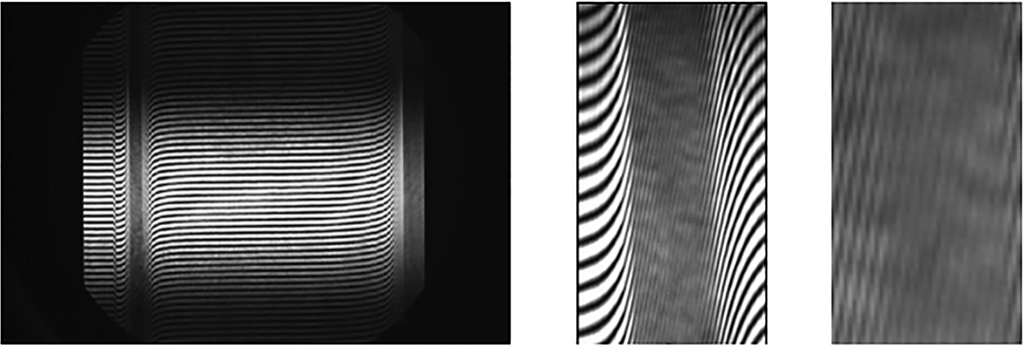
Interferometric image of a highly concentrated protein solution (147 g/L) during centrifugation. (middle and right) Image enlargements of the sedimentation boundary region.

The primary result of an AUC interference scan is an interferogram registered on the CCD camera of the optical system. In the case of Aida, this is a 2672 • 4008 pixel greyscale image. The image shows a black and white interference pattern which is shifted upwards with increasing refractive index within the concentration profile. For high concentrations, these shifts can be extremely steep. A typical image, including a zoom into the boundary, is depicted in Fig. 2. Transformation of this image into usable data is performed by Fourier transform of every pixel column—the phase shift from one column to the next is the information required, and it is directly related to the local concentration difference by the following relation

$$dc=d\phi \cdot \lambda \cdot \frac{1}{l\frac{dn}{dc}}$$ (2)

where d_φ_Φ is the phase shift and dc the corresponding concentration difference. The optical pathlength through the cuvette *l*, the laser wavelength λ and the refractive index increment are considerchemical composition of the analyte in questioned constants for a defined experimental setup and the chemical composition of the analyte in question.

For best Fourier transform performance, it is convenient to adjust the vertical image range to an integer multiple of the range for a single phase, i. e. a black and white stripe in terms of image pixels. Otherwise, noise can develop. The optimal window size and offset for a given number of interference fringes was determined from an optimizing procedure on an initial image of an experiment. Typically, we used 20–30 visible fringes covering 50–60 ppf (pixels per fringe) in the current setup. These Fourier window dimensions were then used for transformation of all pixel columns. It is crucial that this value is valid for all columns, and if the camera is aligned perfectly to the two beams coming from the light source, all fringes will actually cover the same number of vertical pixels, regardless their steepness. Though fringes inside the boundary appear much narrower, they have the same thickness in the vertical direction.

**Fig 3:**
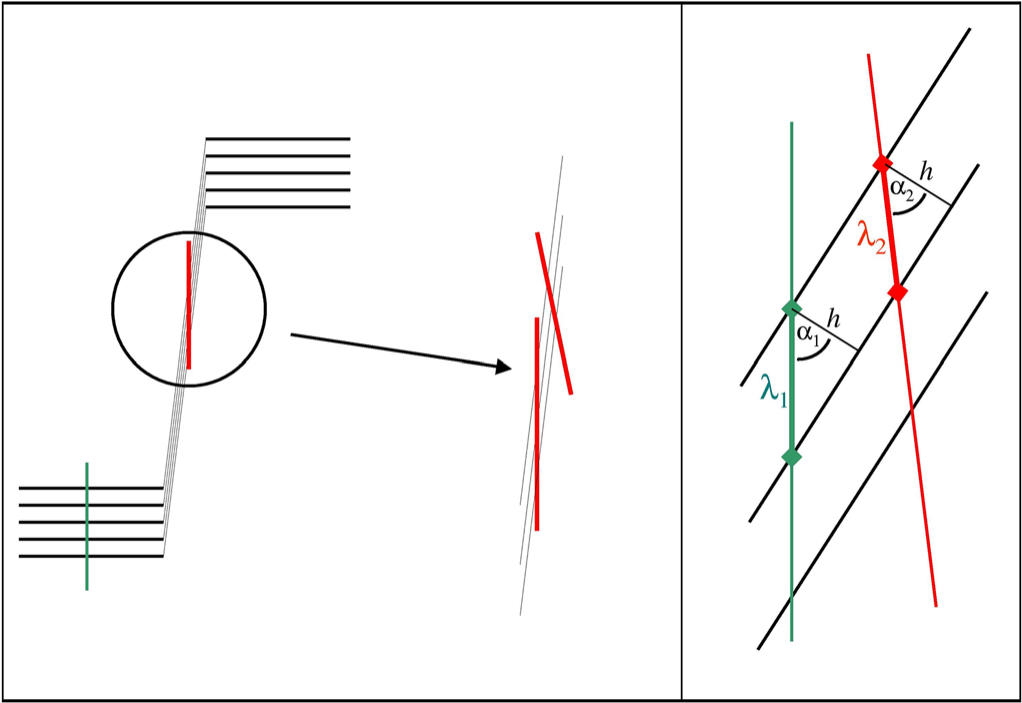
The crucial parameter pixel per fringe (ppf) is not identical for boundary and plateau regions if the camera is not aligned perfectly.

However, if the camera is not aligned perfectly, it is obvious that a line of the same length will cross more or less fringes in the steep boundary region in comparison to the plateau region. Fig. 3 illustrates how a slightly tilted line will cross more fringes in the boundary region. Correspondingly, the ppf value will decrease. The effect is quantified by the relation

cosα1cosα2=λ2λ1 (3)

where α_1_ and α_2_ are the interception angles for the ideal and the erroneous case and λ are the wavelengths in terms of ppf, as given in Fig. 2. Thus, Equation 3 allows for calculation of the camera angle error if the fringe wavelengths have been determined inside the boundary and in the plateau region and if α_1_, the steepness of the fringes in respect to the baseline, is known.

**Fig 4:**
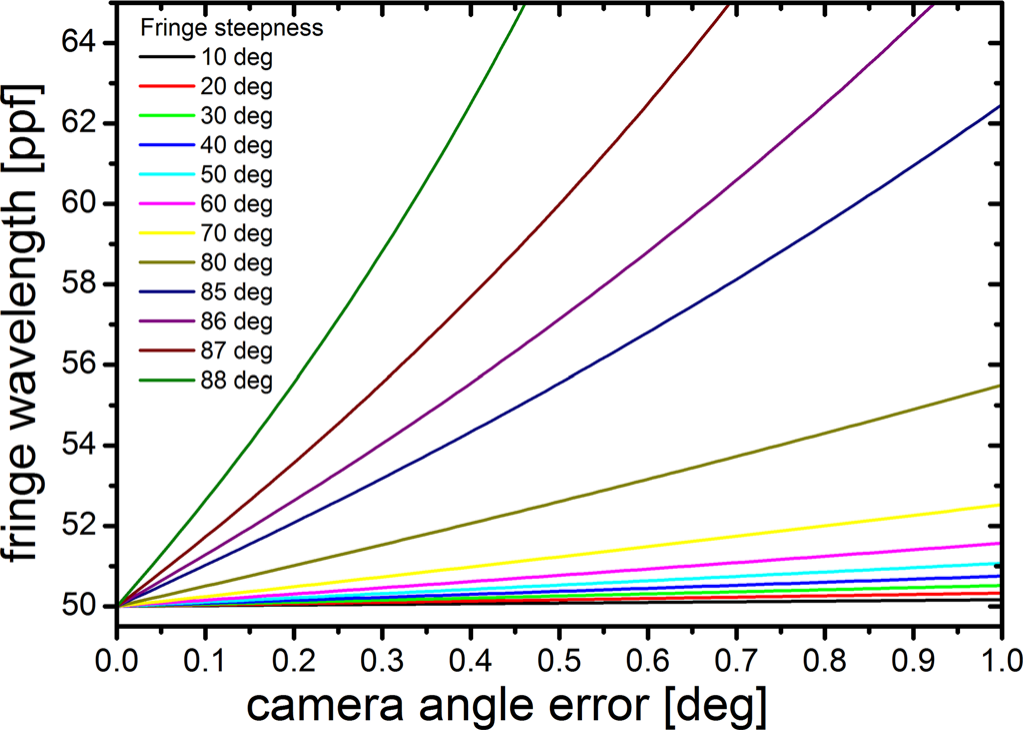
For steep fringes, their wavelength is strongly dependent on camera alignment error. Simulated wavelengths, dependent on camera error and fringe steepness, based on a plateau wavelength of 50 pixel per fringe (ppf).

We calculated the situation for a number of cases for more or less concentrated solutions. Fig. 4 shows that for fringe steepnesses above 80°, as they are encountered for highly concentrated solutions, camera alignment errors far below 1° cause wavelength errors well above 10%. In practice, we found that errors up to 5% can be tolerated; if they are larger, the inner region of the boundary will not be transformed properly. In fact, the most precise method of alignment is to run a concentrated sample and to rotate the camera during the experiment until a maximum number of fringes is transformed within the boundary. In this manner, we have achieved camera alignment errors below 0.05 degrees.

In summary, measurements at high concentration require a maximum of perfection in camera alignment, and there is no mathematical means of correction leading to results of comparable quality.

### Run conditions: Increase of diffusion spreading via a comparatively low rotational speed

It is common practice to perform sedimentation velocity experiments at angular velocities as high as possible. Besides keeping experimental time short for economical reasons, the main motivation is to minimize diffusion broadening and smearing of the sedimentation boundaries established during the experiment. Under nearly ideal sedimentation conditions, the most important barrier for a correct interpretation of a sedimentation velocity experiment is the boundary spreading caused by diffusion (e.g. [24]).

High angular velocities, intuitively understood as a means of performing the experiment rapidly, allow less time for diffusion broadening and apply as much centrifugal force as possible for fractionation of the species present. This can be demonstrated with the equation of Van Holde (4) that approximates the shape of the boundary to the inverse Gaussian error function Φ^-1^. The fraction *w* in the argument of ϕ is a value between 0 and 1, 0 indicating the bottom of the boundary and 1 its upper plateau (i.e. no spreading occurs in the center of the boundary where *w* is 0.5).

$$s_{w}^{*}=s-\frac{2\sqrt{D}}{r_{m}\omega^{2}}\Phi^{\text{-1}}\left(\text{1-2}w\right)\frac{\text{1}}{\sqrt{t}}$$ (4)

Besides the fact that diffusion broadening occurs with √*t* (whereas sedimentation scales linear with *t*), we also encounter the angular velocity ω (squared!) in the denominator, so a high angular velocity drastically reduces broadening of *s* to *s*_*w*_ * at any boundary fraction. On the other hand, diffusion broadening may reduce the steepness of the fringe gradients as the major technical challenge for measuring highly concentrated protein solutions.

We addressed the impact of the rotational speed and found that promoting diffusion broadening significantly enhances an accurate quantification of populations with distinct sedimentation coefficients. With a typical boundary width of 0.5 mm, a 150 g/L antibody solution would exhibit an average fringe steepness of 1270 fringes/cm at a rotational speed of 40 krpm.

**Fig 5:**
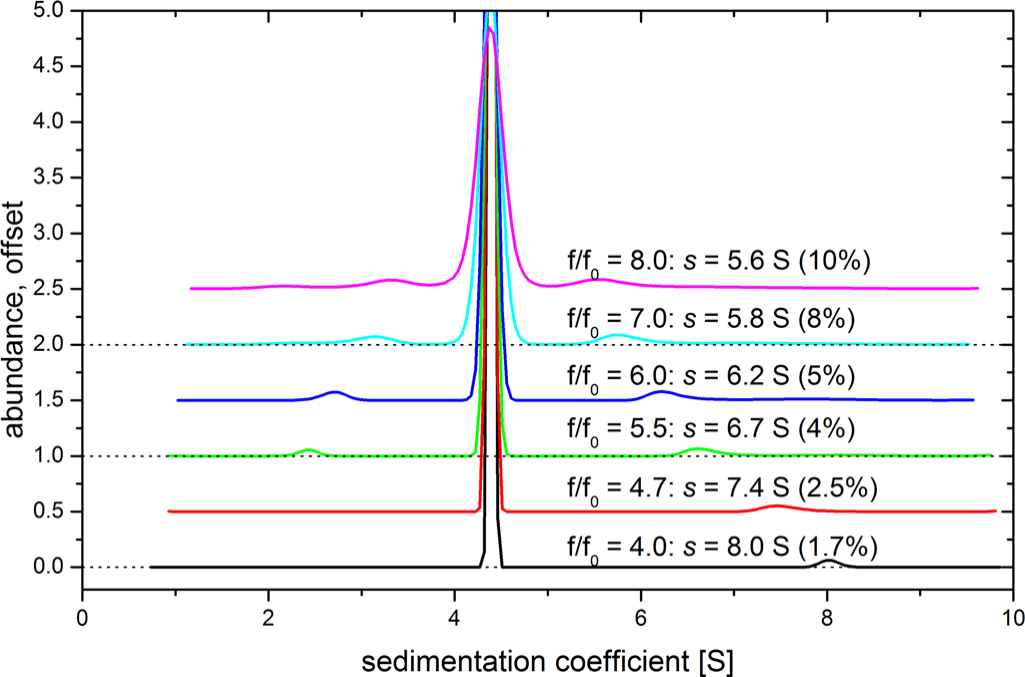
Influence of the frictional ratio on a global fit. Sedimentation velocity experiment of antibody was conducted on the Aida ultracentrifuge at 25,000 rpm, 20°C. The c(s) distributions of data fits applying different frictional ratios are shown. Sedimentation coefficient and relative content of putative dimers are indicated.

Decreasing the average slope of fringes by increasing diffusion broadening provided more usable data, a smaller zone demanding for interpolation and more nodes in the outer boundary for interpolation. As detailed below, the improvements on data interpolation (which remains necessary in the boundary center) were drastic when choosing the much lower angular velocity of 25 krpm for the experiments on antibody 1 at 147 g/L. The fit retrieved the expected signal amplitude in high precision, while sedimentation coefficients and the signal for minor species were not affected significantly at different angular velocities (Fig. 5). Importantly, retrieving the correct total signal is essential for converting the signal of a minor species into the correct percentage of the total signal, i.e. its relative content. If the total signal is found too low, a relative percentage will be calculated too high.

### Enhancements in data analysis

#### Evaluation: apply uniform input data scope

Common rules defining a uniform data scope to fit were applied to all data leading to reproducible results. The data scope encompasses the first and the last scan of an experiment to be incorporated into the fit. In general, initial scans where the boundary has not yet cleared the meniscus and obsolete scans at the end of the experiment were discarded.

Special care has to be taken when defining the range of target sedimentation coefficient values for fitting. Usually, it is reasonable to extend the range to sedimentation coefficients as high as they are covered by the experiment. For highly concentrated systems, however, we discovered that the fitting algorithm tends to create artificial populations at higher sedimentation coefficients, placing some of the total material there with small rmsd contributions. It appears that their impact on error is small in that region, compared to the sedimentation boundary where the extreme steepness of the boundary (due to hypersharpening) produces most of the error residuals in a fit based on the assumption of ideality. In order to block this unintended behavior, we cut off a range of target values where no species are to be expected.

The same applies to the range of sedimentation coefficients between 0 and 0.2 S. Here, no material is to be expected neither, and better fit results were obtained when constraining the fit to a region above 0.2 S.

#### Evaluation: float frictional ratio

The non-ideal nature of highly concentrated solutions raises the question of how to deal with the frictional ratio f/f_0_, which is an important fitting parameter. This factor connects the sedimentation coefficient and the mass of an object, expressing its frictional properties. It thus substitutes the diffusion coefficient D by means of the Einstein equation D = kT/f in the Svedberg equation (5):

$$M=\frac{sRT}{D(1-\bar{\nu }\rho )}$$ (5)

The frictional ratio is a value larger or equal to 1, giving the factor by which the frictional properties are increased in respect to a compact rigid sphere of same mass and density. A compact sphere with a smooth surface represents the object with smallest possible frictional resistance *f*_*0*_ and, thus, most rapid sedimentation *s*_*0*_.

Modern sedimentation velocity analysis allows for fitting of *f/f*_*0*_. In fact, this is instrumental for the c(s) method; the necessary information is given by diffusion broadening of sedimentation boundaries throughout the experiment. The frictional ratio can be described by the following relation [25, 26]:

$$\frac{f}{f_{0}}=\frac{s_{0}}{s}$$ (6)

By means of Equation 5, the molar mass of any population can be calculated from its sedimentation coefficient under ideal conditions. However, highly concentrated protein solutions are highly non-ideal, which not only decreases the rate of sedimentation but also affects boundary shape which is less broadened by diffusion than it should be, according to ideal hydrodynamics.

Hypersharpening of sedimentation boundaries is a typical feature of sedimentation boundaries for highly concentrated solutions, as in the current study. Due to unspecific interparticle interactions, particles sediment slower in highly concentrated solutions. A sedimentation boundary can be understood as an interface between a low concentrated solution (depleted of particles) and a highly concentrated solution (loaded with the initial concentration). In consequence, particles in the front of the boundary will be stronger exposed to a reduction in velocity as trailing particles. While the leading particles are engaged by the sedimentation boundary, the trailing particles catch up—both processes make the boundary steeper.

Hypersharpened boundaries can often be identified by visual inspection, as in the present case. This provides evidence for non-ideal behavior. However, the Lamm equation (1), upon which the fitting process is based, does not comprise interparticle interactions. Thus, the steep boundaries are dedicated to the other transport process—diffusion—contained in the Lamm equation. Diffusion initiates boundary broadening, whereas sharpening as the opposite process is equivalent to slow diffusion. In consequence, the fitting program obtains large frictional terms, resulting in low diffusion and sharp boundaries, thus doing its best to reflect the boundary shape in the set of hydrodynamic parameters available.

This allows the fitting program to present reasonable fits despite the fact that non-ideal sedimentation is not part of its parametrization. Though a more reasonable value for *f/f*_*0*_ can be estimated (or taken from fits at diluted conditions and, thus, ideal conditions), we found that allowing the fitting algorithm to accomodate non-ideality in the frictional ratio produced a decent fit, yielding robust information on sedimentation coefficients and abundances.

The best option proved to be a floating *f/f*_*0*_, even though the fit result does not describe a consistent model of *s*, *M*, and *D*. Thus, we enabled the fit to reduce error squares on a more sensitive level, accepting an unrealistically high frictional ratio. Since *f/f*_*0*_ was obtained much too high for the reasons given above, molar masses calculated from sedimentation coefficients (Equation 5) became obsolete. This did not hamper the interpretation of the results, since quantitification of populations as the crucial result was robust. Furthermore, the true molar masses of antibody monomers and dimers could be easily determined via a concentration-dependent analysis as shown below.

Though floating the frictional ratio in order to achieve a best fit to the experimental data, far off from the actual value, has been shown to provide the best results possible, there is another consequence arising from the fact that *f/f*_*0*_ is governed by hypersharpening rather than by the hydrodynamic properties of the molecules: if experiments are conducted at different angular velocities on the same system, hypersharpening can be differently prominent. As outlined above, a lower than usually employed rotational speed turned out to be crucial in handling extremely steep fringe gradients. Consequently, fits on these experiments will yield different frictional ratios. This, in return, might affect the fit, especially regarding minor populations in the presence of dominating species that are not physically fractionated from one another during the experiment.

Fig. 5 demonstrates that different frictional ratios applied to the same velocity experiment produce some fluctuation in respect to the putative dimer to the right of the main peak. It is displaced, and its area decreases with smaller *f/f*_*0*_. Also, a non-proteinaceous population left of the main peak, presumed to be artificial, diminishes. This experiment was conducted at 25 krpm, and the fitted frictional ratio was 4.71. Another experiment on the same system, but at 40 krpm yielded a frictional ratio of 8. The dimer’s sedimentation constant at 40 krpm was found to be 5.5 S, close to the value of 5.6 S found at 25 krpm using the”wrong” frictional ratio of 8 (Fig. 5, uppermost data). However, the relative area for this peak is found nearly identical if the individual *f/f*_*0*_ is used. The raw data, data fits and residuals to the c(s) distributions shown in Fig. 5 are depicted in S1 Fig.

Comparison with unpublished data of another antibody with a much smaller aggregate content than found in the formulations characterized in the present study revealed that the stability of a minor species towards modifications in the frictional ratio increases significantly if the species is present with more than 2% of the total signal. Also, it should be noted that regardless the displacement of the peak, the crucial information, its area, is remarkably stable even at very low dimer contents. Thus, we conclude that the influence of the frictional ratio on the fit is not a concern.

#### Evaluation: interpolate steep boundary region

**Fig 6:**
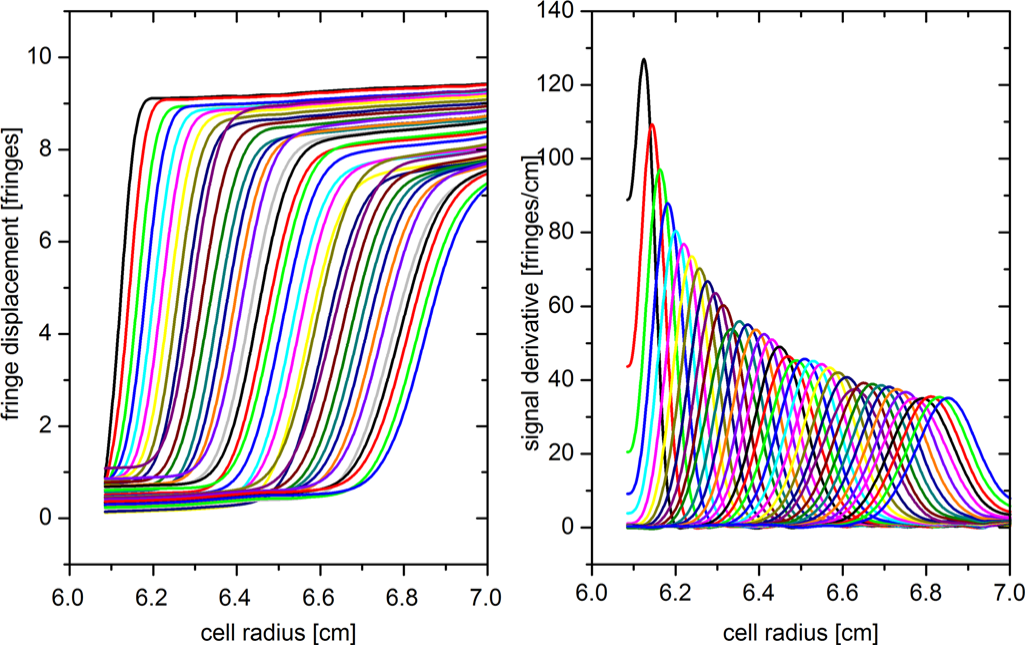
Typical interference data for a sedimenting single species. The derivatives on the right resemble a Gaussian.

We have discovered an unusual phenomenon while analyzing SV data recorded at high solute concentrations. At moderate concentrations, a monomodal, monodisperse solute will exhibit a sedimentation behavior as shown in Fig. 6. The shape of the boundary is well resembled by a Gaussian error function; correspondingly, the derivative is approximated by a Gaussian with a second moment, meaning that the peak is slightly tilted to the left. In fact, it has been common practice for decades to localize the second moment of each scan and to calculate the average sedimentation constant from its movement with time [27–30], before modern computing allowed for finite element fits of the entire scan data.

**Fig 7:**
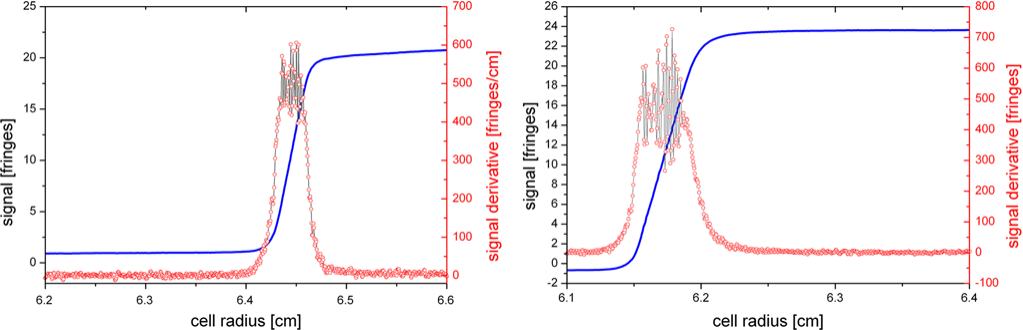
Two examples for highly concentrated samples at different run conditions. Though the signal derivative should be a Gaussian, the signal increases with a nearly constant slope in the center of the boundary. The signal derivative does not exceed 500 fringes/cm.

We have observed, however, that fringes inside the boundary for highly concentrated solutions do not exceed a steepness of 500 fringes/cm. Though the fringes are continuous and appear normal by visual inspection, they in fact do not follow the Gaussian error function but rather exhibit a linear slope in the center of the boundary. Two examples are shown in Fig. 7. Correspondingly, the total signal does not attain the theoretical value given by Equation 2, scaling strictly linear with concentration at constant optical pathlength, laser wavelength, and refractive index increment.

The observation that we are able to record well resolved interferograms for steep concentrations, yet the fringes do not follow the expected shape for a boundary suggests that we encounter optical limitations. A nearby theoretical approach is to consult Svensson's equation (7) describing second order aberrations in the interferometric measurement of concentration gradients [20, 21]:

$$\Delta S=l\left(n-n_{0}\right)+l^{2}\beta \frac{dn}{dr}\frac{1-2\text{r}}{2n_{0}}+l^{3}{\left(\frac{dn}{dr}\right)}^{2}\frac{2-3r}{6n_{0}}$$ (7)

where Δ*S* is the phase shift between sample and reference beam in terms of a length, *l* is the optical pathlength of the measurement cell, β the incident angle in respect to the optical axis, *n* the refractive index of the sample at a given position, d*n*/d*r* the refractive index gradient at that position, *n*_*0*_ the reference's refractive number, and *r* the position of the focal plane within the cell in terms of a fraction of *l*, 0 < *r* < 1.

It is apparent that we need not consider the second term as the vast majority of light enters the cell parallel to the optical axis. However, the third term requires consideration as the (steep) refractive index gradient occurs squared. Lloyd [21] has calculated the third term to become observable at refractive index gradients above 0.004/cm, which corresponds to a signal of approximately 67 fringes for a 1-cm-cell, depending on laser wavelength. "Observable" means a contribution of 0.02 fringes, which is the achievable precision for the interference system the author gives elsewhere. It should be noted, however, that the contribution specified is minor in respect to the total signal; on the other hand, the concentration gradients in our experiments, attaining 800 fringes/cm, exceed the limit given by Lloyd [21] by more than one order of magnitude. This effect is opposed by the facts that (a) we have used 1.5-mm-cells rather than 10-mm-cells (*l* occurs in the third power, drastically reducing the third term) and (b) Lloyd [21] is assuming a focal plane of *r* = 0.5 which was common at that time, whereas our system, alike all interference systems nowadays, is focussed to *r* = 2/3 which theoretically should make the third term vanish. The critical focus on the 2/3 plane is achieved with the aid of a calibration cell with a sharp pattern at the desired position inside a standard cell housing. For other pathlengths (3 and 1.5 mm), this focus is maintained as we use custom made housings that guarantee the same position for the 2/3 plane in the shorter centerpieces. Though the relative error of the focus plane will increase when coming to shorter pathlengths, this effect is conveniently opposed by the factor *l* ^*3*^ in the third term of the Svensson equation, making this term smaller by a factor of 512 for the transition from 12 mm to 1.5 mm optical pathlength. In fact, the relevant factor would be 64, which results from the relation (*l* ^*3*^ vs. *l)* to the first term.

**Fig 8:**
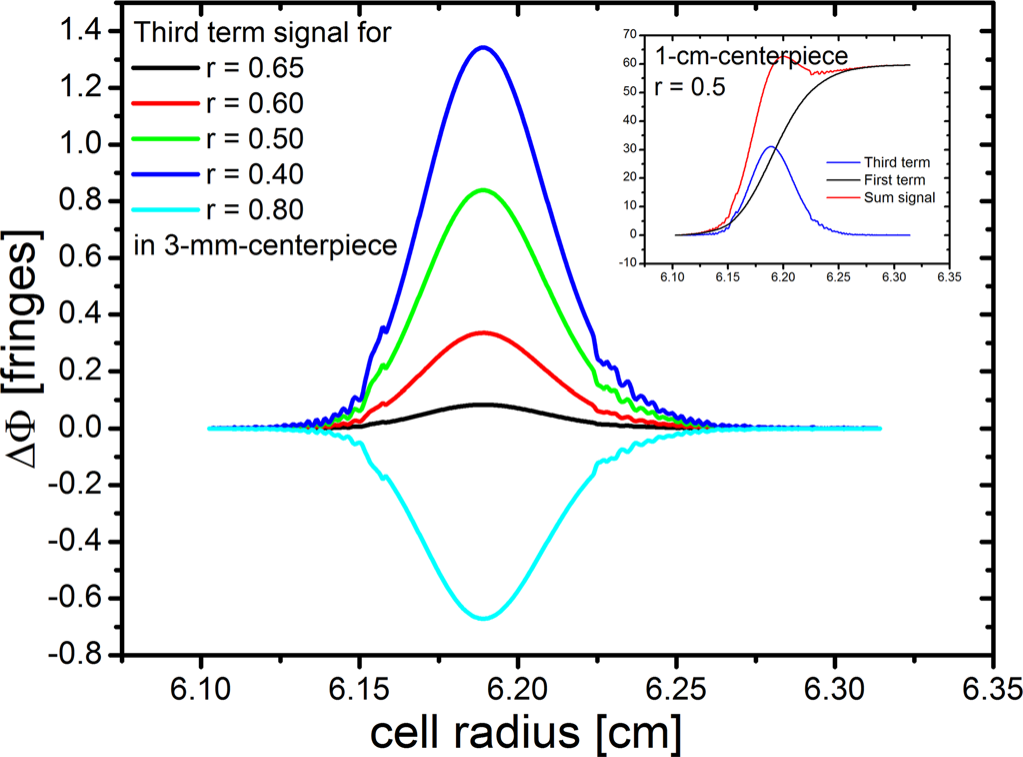
Simulation of the impact of the third term of the Svensson equation (7) for a typical dataset from this study for different values of *r*. For easier interpretation, we have transformed ΔS into fringes, using our laser wavelength of 660 nm. The plot shows that in the center of the boundary, at a refractive index gradient of 800 fringes/cm, a contribution of 1 fringe to a total signal of approximately 80 fringes is attained only for a vast error in *r*. The inset shows a comparison for 1-cm-cells at the same gradient with a much larger impact and clearly visible Wiener skewing of the total signal.

To demonstrate the influence of an error in *r*, we have simulated the impact of the third term of Equation (7) for a typical dataset from this study for different values of *r*, as depicted in Fig. 8. For easier interpretation, we have transformed ΔS into fringes, using our laser wavelength of 660 nm. The plot shows that in the center of the boundary, at a refractive index gradient of 800 fringes/cm, a contribution of 1 fringe to a total signal of approximately 80 fringes is attained only for a vast error in *r*. The inset shows a comparison for 1-cm-cells at the same gradient (which would require a 6.7fold diluted solution) with a much larger impact and clearly visible Wiener skewing of the total signal.

This simulation clearly shows that our observation that a gradient of 500 fringes/cm is not exceeded cannot be dedicated to the formation of the interferogram as described by the Svensson equation (7) for three reasons: (a) the third term causes skewing of the boundary but does not reduce the total signal as observed in our study, (b) the typical skewing is not observed with our data, rather exhibiting a linear trend, (c) the sensitive procedure of focussing should cause a variation in *r* and, thus, the observed effect after occasional realignment of the instrument (where we observe the very same limit anytime), and (d) the interferogram produced according to the Svensson equation should be continuous, also in the first derivative, which is not what we observe.

We believe, therefore, that the limit we encounter is simply a consequence of optical resolution. If we model a single light beam projected from the object plane to the image plane, we calculate that an infinitely small light source will exhibit a diameter of 40 μm on the image plane. This means that two distinguishable objects need to be at minimum 40 μm apart on the object plane. Considering our magnification factor of 2, the distance is reduced to 20 μm, and associating this distance with our unit of 1 fringe, we obtain a maximum fringe gradient of 50 fringes/mm, which is in fact the limit we observe.

Therefore, this limit can only be overcome by a larger magnification factor, requiring a larger projection plane. We intend to realize this approach in the future. In comparison to the XL-I system with a magnification factor of approximately 1.75, our factor of 2 should enhance the gradient limit by 30–35%, meaning that the XL-I should fail to image gradients steeper than 375 fringes/cm. In practice, however, the XL-I system is observed to fail at lower gradients. There are three reasons for the better performance of the novel system: (a) the higher camera resolution provides more datapoints for Fourier transform, (b) the larger number of depicted fringes stabilizes Fourier transform under difficult conditions, (c) the optimization of the Fourier window has a considerable effect especially for steep fringes.

In order to”repair” the data and enable data evaluation, we decided to interpolate the inner boundary. We have chosen the following function (Equation 8) which has been shown to well describe the derivatives of intact boundaries, measured at moderate concentrations:

$$y=y_{0}+Ae^{-e^{-z}-z+1},\;z=\frac{x-x_{c}}{w}$$ (8)

Here, *y* is the derivative signal in fringes/cm, *y*_*0*_ is the ordinate offset, *x*_*c*_ the center of the peak, *A* its height, and *w* its width. This is simply a function describing the inner boundary shape; there is no physical interpretation of the parameters. We replaced the questionable datapoints within the boundary by fitting function (Equation 8) into the truncs of the boundary’s derivative, cut off at typically 350 to 450 fringes/cm, and reintegrating.

**Fig 9:**
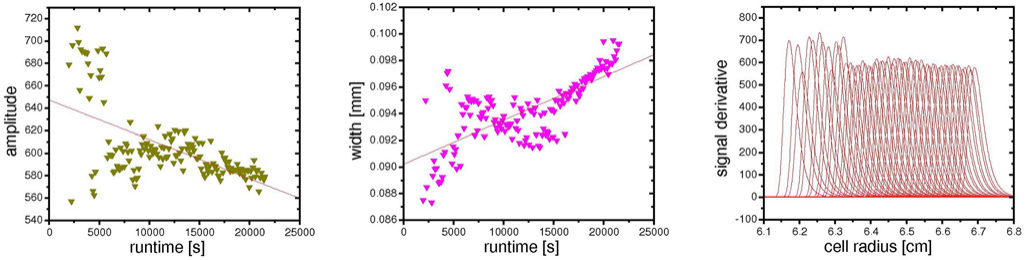
Fit results for peak height and width, and entire peaks created from the individual fitting parameters. These results exhibit some noise; optional linearization would force the parameters onto the red lines, providing a cleaner interpolation.

We took advantage of the large number of result parameter sets by fitting global trends for *y*_*0*_, *A*, *x*_*c*_, and *w* throughout all scans of an experiment. As can be seen in Fig. 9, there is some individual variance for the fit of each scan. As it is apparent that some trends occur, e.g. an increase of width due to diffusion broadening or a decrease in peak height and area due to radial dilution, it is justified to imply a continuous trend for these parameters. Therefore, the uncertainty of each single fit can be decreased by aligning the fit results onto the global trend.

A useful collateral result of the fitting process is the dataset of second moment peak center values. These can be analyzed according to the classical second moment vs. runtime integral plot (see below), yielding a sedimentation coefficient that should be retrieved in the subsequent global fitting process and a loss integral referred to below.

We found that the suggested fitting function yields the expected area (equivalent to the total fringe displacement inside the boundary) for protein concentrations up to 75 g/L when run at 40 krpm. At a rotor speed of 25 krpm, the expected integral was found even for protein concentrations up to 147 g/L. The interpolation method encountered its limitations only for measurements on high concentrations at high angular velocities. At 40 krpm, the boundary attains its limited steepness so quickly that not enough nodes at the truncs of the derivative are present to stabilize the interpolation function properly.

Though an additional constraint can be introduced into the fitting procedure, forcing the area to the expected total signal, we consider this a too invasive manipulation of the raw data. We rather respect the limits of this interpolation, accepting the applicability of this procedure as well. Thus, for data evaluation of highly concentrated antibodies above 20 g/L, the interpolation was allowed to float freely, resulting in total signal intensities obtained too low for solute concentrations larger than 75 g/L if run at angular velocities higher than 25 krpm.

#### Evaluation: second moment method for average sedimentation coefficient

In general, the sedimentation coefficient of any species present at the radial position r of the concentration profile in a scan taken at t seconds after the start of the experiment can be calculated by the simple transformation

$$s={\frac{\ln\frac{r}{r_{m}}}{\int \omega^{2}dt-\left(\omega^{2}t\right)}}_{loss}$$ (9)

without any assumptions but the geometry of the experiment. *r*_*m*_ is the radius of the meniscus; ∫ω^2^d*t* is the runtime integral, calculated from the angular velocity ω at any time of the experiment integrated over the time during which it was valid. (ω^2^ *t*)_*loss*_ is the loss integral, an additional amount of energy required in order to overcome surface tension at the solvent interface, detaching molecules from the meniscus.

Though the dimension of the runtime integral is a reciprocal time and the typical unit is given in GHz, it should not be understood as a frequency, but as a measure for the total centrifugal power the system has been exposed to from the beginning of the run until the time at which the scan was acquired.

The classical method for calculating average sedimentation coefficients is therefore a plot of ln(*r*_*bnd*_ */r*_*m*_) (*r*_*bnd*_ being the second moment center of the boundary) vs. runtime integral. It should yield a straight line of slope *s*, passing through the origin: at a runtime integral of zero, *r*_*bnd*_ should be equal to *r*_*m*_, as no sedimentation has yet occurred. Taking the loss integral into account, the line does not intersect the abscissa at *ω* ^*2*^ *t* = 0, but at a positive value.

**Fig 10:**
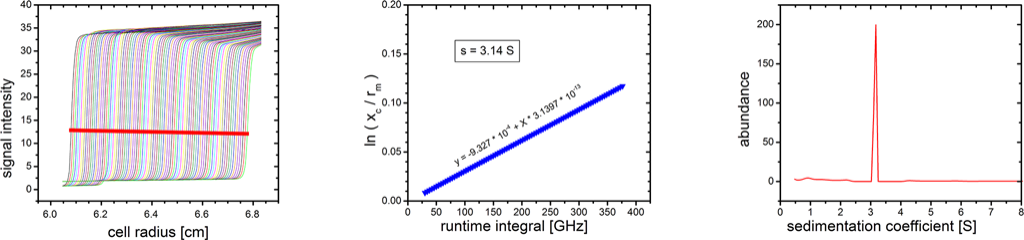
Average sedimentation coefficient from second moment method. Sedimentation boundary second moment position as calculated in Results (left), regression plot on runtime integral (center) yielding s = 3.14 S. This coefficient should match the peak obtained from global finite element fitting (right).

As our interpolation procedure (see below) involves calculation of *s*_*bnd*_ for each scan, it is feasible to associate the second moment method, offering three important options:

1. Smoothing the *x*_*c*_ values as obtained from the fit according to Equation 8 rather than linear,
2. Obtaining a value for *s* that should be retrieved in global fitting as shown in Fig. 10; this match is an important verification for an optimized data scope and data range as documented below.
3. Obtaining a loss integral (see below).

Significantly, the final data indeed showed a high level of consistency in respect to globally fitted *s* and *s* calculated by the second moment method. This assures a high level of reliability in respect to the interpolation process.

#### Evaluation: loss integral correction

According to Equation 9, we subtracted the loss integral from the recorded runtime integral prior to data evaluation. We received fits of high quality and also observed that allowing the meniscus to float during the fitting procedure did not yield a value significantly different from the actual meniscus position. This behavior is different for non-compensated data.

**Fig 11:**
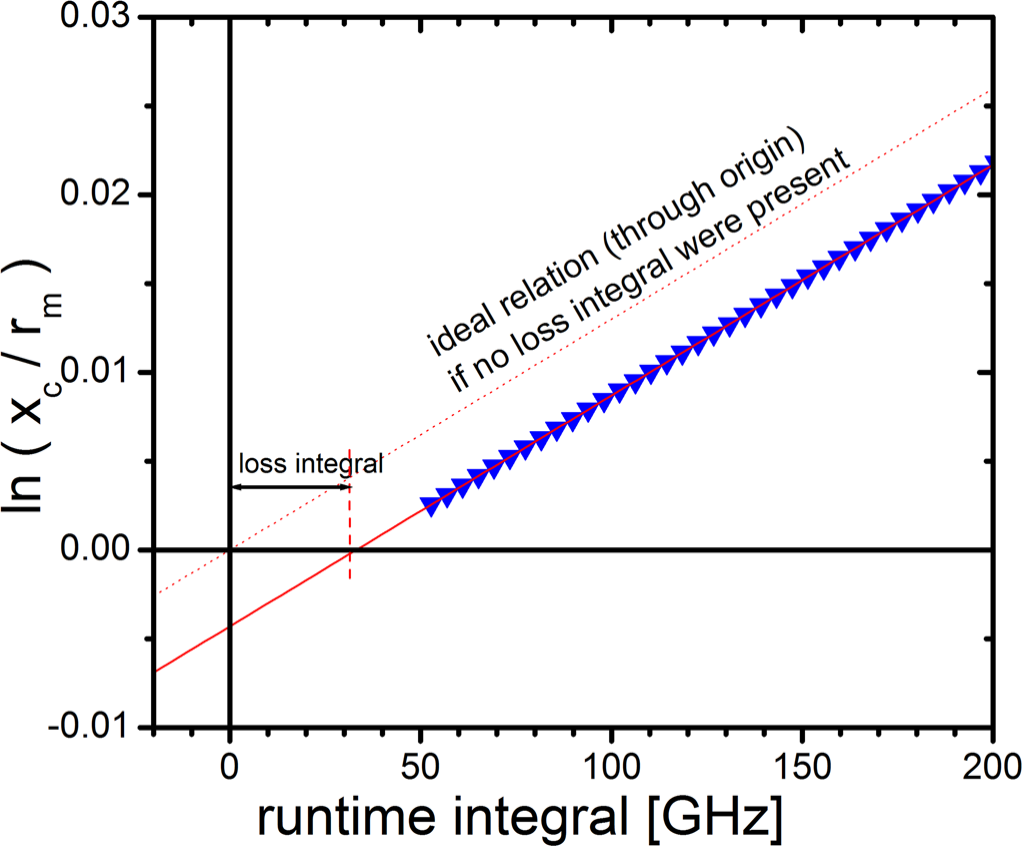
Plot of ln(r_bnd_/r_m_) vs. runtime integral revealing a loss integral. A discrete amount of centrifugal force has not contributed to displacement of the sedimentation boundary, but was needed to overcome surface tension at the meniscus.

Fig. 11 illustrates the procedure we followed for determination of the loss integral for each individual experiment. It shows an ln(*r*_*bnd*_ */r*_*m*_) vs. *ω* ^*2*^ *t* plot for real data. For *r*, we used *x*_*c*_, the center of the tilted Gaussian fit according to Equation 8 for a number of scans. Following Equation 9, this plot yields a straight line with the slope *s*, passing through an abscissa intercept larger than zero. This is the loss integral, needed to detach material from the meniscus, but not causing a displacement of the sedimentation boundary.

We found that for diluted solutions and for experiments conducted at high angular velocities as 40 krpm, the loss integral is typically in the magnitude of several Ghz. However, at high concentrations (147 g/L) and moderate angular velocities (25 krpm), the loss integral was consistently in a range from 25 to 35 GHz, thus having a significant impact on the reproducibility of results and on the precision of control parameters (fitted meniscus radius, frictional ratio, total signal). We compared the extent of the loss integral for different experimental situations, in particular its dependence on solute concentration and angular velocity. The following trends are observed and may be generalized: 1. The effect increases with solute concentration. 2. The effect decreases with angular velocity.

### Sedimentation velocity of undiluted antibody 1 at 147 g/L measured at low rotational speed of 25 krpm

The measurement and data evaluation was performed according to the experimental design and evaluation procedure developed during the course of this study as outlined above.

Table 1 displays fit results for four replicates on antibody 1. Two results were specifically outstanding. First, the detected signal amplitude matched the expected signal perfectly. This is seen in the experimental refractive index increment as calculated from the given concentration, the laser wavelength, the optical pathlength, and the total fringe displacement of averaged 64.8 fringes. Thus, d*n*/d*c* was found to be averaged 0.194 mL/g, representing a typical value for globular proteins [30]. This indicates a near quantitative mass retrieval in agreement with lacking evidence for any significant precipitation leading to a pronounced recovery loss. The approximately fully recovered protein amount after dilution (see below) confirms a nearly quantitative mass retrieval.

**Table 1:** Sedimentation velocity of antibody 1 at 147 mg/mL measured at 25 krpm.

run	f/f_0_	rmsd	signal	dn/dc	loss integral	monomer		dimer		oligomers
		[ΔΦ]	[ΔΦ]	[mL/g]	[GHz]	[S]	[%]	[S]	[%]	[%]
1/3	25.69	1.869	64.83	0.194	33	1.33	92.1	2.18	2.6	5.3
1/4	24.66	1.743	62.66	0.188	35	1.36	92.4	2.15	2.5	5.1
1/5	24.74	1.786	66.39	0.199	23	1.36	90.0	2.18	3.2	6.8
1/6	24.78	1.459	65.25	0.195	29	1.36	90.7	2.03	3.1	6.3
m	24.97	1.714	64.78	0.194	30	1.35	91.3	2.14	2.8	5.9
SD	0.48	0.18	1.56	0.01	5	0.02	1.1	0.07	0.3	0.8

The optimized experimental and data evaluation protocol was used. Total signal and rmsd are given in units of fringe displacement. The refractive index increment d*n*/d*c* is calculated from the total signal as an indication of mass retrieval.

Second, the fit results obtained for the four replicates are in excellent accordance. In particular, it is striking that the highly disturbed parameter *f/f*_*0*_, allowing the fit to adapt to non-ideal conditions far away from the prerequisites of the Lamm equation (1), was obtained in such good reproducibility. The actual frictional ratio of these antibodies has been found to be about 1.7 under approximately ideal conditions (see below).

**Fig 12:**
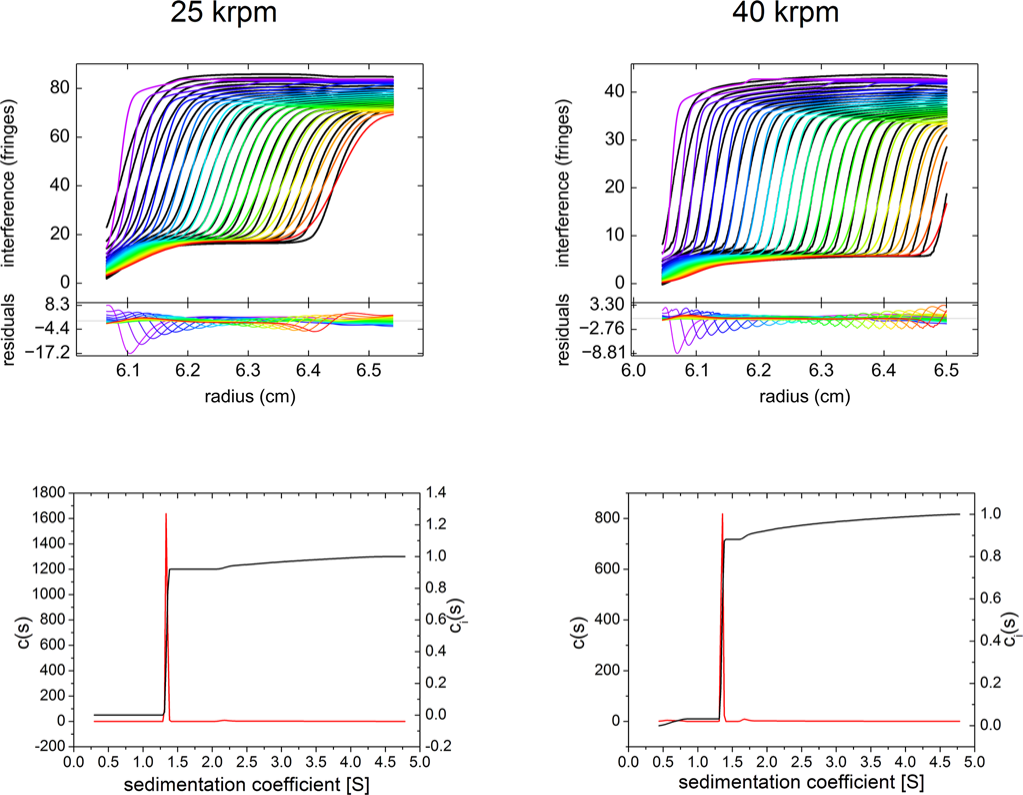
Example of c(S) analysis of undiluted antibody 1 at 147~mg/mL using data aquired with the Aida interference detector. Sedimentation velocity experiments were conducted at 25,000 rpm, 20°C (left) and 40,000 rpm, 20°C (right), respectively. The upper top panels show raw data (circles) and best fit (lines). For clarity, only every fifth scan of the data set is shown. The lower top panels show best fit residuals of the plotted scans. The bottom panels show the c(s) distribution.

The comparatively high rmsd were moderate in respect to the high total signal (2–3%). This indicates the fit to be highly reliable, succeeding in allocating most of the non-ideality to the frictional ratio and producing a fit satisfactorily reflecting measurement results (Fig. 12, left top panel). Most of the residuals are located in the region of the main boundary representing sedimentation of monomers.

In line with this, the sedimentation coefficients derived from the superposition of Lamm equation solutions (Table 1) were corroborated by second moment method calculation of average sedimentation coefficients (data not shown). This observation is consistent with previous findings suggesting that ideal transport models can faithfully capture the sedimentation coefficient of a non-ideal boundary, although the time-dependent spreading of the boundary is not reliably modeled [29, 31, 32].

The loss integral, given in Table 1, is discussed above in a separate section. Taking the loss integral into account contributes to the excellent quality of this analysis.

In summary, the monomer content of antibody 1 at a concentration of 147 g/L was found to be 91.3±1.1% applying the optimized experimental and data evaluation procedure. The remaining amounts represent dimers and higher oligomers not larger than tetramers and hexamers, respectively, as assessed from the maximum sedimentation coefficients which are lower than the fourfold value of the respective sedimentation coefficients of monomeric antibodies. For globular proteins a twofold sedimentation coefficient roughly corresponds to the threefold molar mass, i.e. trimers are expected to have the double sedimentation coefficient of the monomeric protein. Due to their low abundance in diluted antibody solutions, the oligomeric state of aggregates larger than dimers could not be verified by the concentration-dependent analysis. In contrast, the identity of monomers and dimers was unambiguously confirmed via the concentration-dependent analysis of diluted antibody solutions exhibiting approximately ideal sedimentation behavior (see below).

Of particular relevance is the Johnston-Ogston effect (J-O effect), a concentration anomaly found in multicomponent systems as the present solution with several antibody size-variants. The effect is caused by the sedimentation of faster components delaying the sedimentation of slower components, leading to an apparent increase of the amounts of the less rapidly sedimenting population with a corresponding decrease in the apparent amount of the faster components [25]. The accumulation of the slow component continues to grow with increasing run time. This effect is negligible at low solute concentrations and always increases with solute concentration. Several approaches to a quantitative analysis have been proposed, which however are restricted to two-component systems [33–38].

Correia et al. [38] presented accurate numerical solutions of the Lamm equation for systems exhibiting the J-O effect, provided that there exists a plateau of the slow component by itself as well as a plateau with both fast and slow component present.

Since these conditions are not fulfilled and more than two size-variants are present, we did not attempt to correct for the J-O effect, which contributes to the visible hypersharpening of the monomer boundary. Visual inspection also confirms the presence of oligomers in the plateau region. Notable is the fact that higher oligomers are found in about twofold amount than dimers (Table 1), though the J-O effect should cause slower sedimenting dimers to be overrepresented vs. more rapidly sedimenting oligomers. Therefore, the J-O effect is comparatively moderate and does not preclude the detection of both dimers and higher oligomers. In line with this, the J-O effect does not lead to a peak (as an extreme hypersharpening) of the main boundary with a subsequent negative concentration gradient before the plateau of more rapidly sedimenting oligomers is formed, as described for systems with strong crossterm interactions [38]. Overall, one can safely assume that the amounts of dimers/oligomers are not overestimated as a consequence of the J-O effect.

### Sedimentation velocity of highly concentrated antibodies measured at 40 krpm

To investigate the impact of the diffusional spread on quality of the raw data and subsequent fit in more detail, we analyzed antibodies 1 and 2 at 40 krpm—both undiluted (147 g/L or 102 g/L, respectively) and after a 1:2 dilution with formulation buffer.

The consistency of the results in respect to frictional ratio, mass retrieval, sedimentation coefficients as well as the relative amounts of found populations is good but significantly lower than those derived from measurements at 25 krpm (Table 2).

**Table 2:** Sedimentation velocities of antibodies 1 and 2 measured at 40 krpm.

antibody 1: 73.5 g/L											
run	f/f_0_	rmsd	signal	signal*	dn/dc*	LMW	monomer		dimer		oligomers
		[ΔΦ]	[ΔΦ]	[ΔΦ]	[mL/g]	[%]	[S]	[%]	[S]	[%]	[%]
4/2	14.91	0.703	33.18	32.58	0.195	1.8	3.12	97.1	3.87	1.5	1.4
4/3	15.15	0.762	33.69	32.98	0.197	2.1	3.15	96.0	3.84	2.3	1.7
4/6	15.24	0.719	33.82	33.16	0.199	2.0	3.15	96.2	3.84	2.0	1.8
4/7	15.52	0.706	34.51	33.27	0.199	3.6	3.14	95.9	3.76	2.1	2.0
m	15.21	0.723	33.80	33.00	0.198	2.4	3.14	96.3	3.83	2.0	1.7
SD	0.25	0.027	0.55	0.30	0.002	0.8	0.01	0.5	0.05	0.3	0.3
antibody 1: 147 g/L											
run	f/f_0_	rmsd	signal	signal*	dn/dc*	LMW	monomer		dimer		oligomers
		[ΔΦ]	[ΔΦ]	[ΔΦ]	[mL/g]	[%]	[S]	[%]	[S]	[%]	[%]
5/1	35.54	0.747	36.51	35.29	0.106	3.3	1.35	87.8	1.67	3.3	9.0
5/3	40.02	0.883	37.75	36.00	0.108	4.6	1.32	86.9	1.57	3.7	9.5
5/4	38.20	0.700	36.35	35.00	0.105	3.7	1.33	83.9	1.57	4.7	11.4
5/7	41.28	0.737	34.75	34.12	0.102	1.8	1.31	86.6	1.55	4.3	9.1
m	38.76	0.767	36.34	35.10	0.105	3.4	1.33	86.3	1.59	4.0	9.7
SD	2.49	0.080	1.23	0.78	0.003	1.2	0.02	1.7	0.05	0.6	1.2
antibody 2: 51 g/L											
run	f/f_0_	rmsd	signal	signal*	dn/dc*	LMW	monomer		dimer		oligomers
		[ΔΦ]	[ΔΦ]	[ΔΦ]	[mL/g]	[%]	[S]	[%]	[S]	[%]	[%]
2/1	8.05	0.444	24.67	23.28	0.201	6.0	3.35	98.7	4.72	1.2	0.1
2/4	8.00	0.442	24.05	22.77	0.196	5.6	3.35	98.9	4.81	1.0	0.1
2/5	7.74	0.431	23.69	22.92	0.198	3.3	3.41	98.3	4.71	1.6	0.1
2/7	8.08	0.451	24.15	22.78	0.197	6.0	3.35	98.9	4.75	1.0	0.1
m	7.96	0.442	24.14	22.94	0.198	5.2	3.37	98.7	4.75	1.2	0.1
SD	0.16	0.008	0.40	0.24	0.002	1.3	0.03	0.3	0.04	0.3	0.0
antibody 2: 102 g/L											
run	f/f_0_	rmsd	signal	signal*	dn/dc*	LMW	monomer		dimer		oligomers
		[ΔΦ]	[ΔΦ]	[ΔΦ]	[mL/g]	[%]	[S]	[%]	[S]	[%]	[%]
3/2	17.75	0.873	35.72	35.71	0.154	0.0	2.00	93.7	2.52	2.9	3.4
3/4	17.69	0.884	35.56	35.57	0.153	0.0	2.02	94.3	2.61	2.3	3.4
3/7	16.69	0.876	35.02	35.02	0.151	0.0	2.04	95.1	2.64	2.1	2.8
m	17.37	0.878	35.43	35.43	0.153	0.0	2.02	94.4	2.59	2.4	3.2
SD	0.60	0.006	0.37	0.36	0.002	0.0	0.02	0.7	0.06	0.4	0.3

Total signal and rmsd are given in units of fringe displacement. The refractive index increment d*n*/dc* is calculated from the total signal after subtraction of non-proteinaceous LMW material as an indication of mass retrieval. Relative contents of monomer and dimer+oligomers are calculated excluding the signal contribution of LMW material.

The following relative contents of antibody monomers were determined: Antibody 1 comprises 96.3±0.5% and 86.3±1.7% monomers at 73.5 g/L and 147 mg/mL, whereas antibody 2 comprises 98.7±0.3% and 94.4±0.7% monomers at 51 g/L and 102 mg/mL, respectively. In contrast to the measurements at 25 krpm, minor populations sedimenting slower than monomers were frequently observed (LMW, Table 2). The remaining amounts represent dimers and higher oligomers not larger than tetramers and hexamers, respectively, as assessed from the maximum sedimentation coefficients which are lower than the 2.5fold value (antibody 2) and fourfold value (antibody 1) of the respective sedimentation coefficients of monomeric antibodies (see above).

Importantly, only the half-concentrated antibodies (51 or 73.5 g/L, respectively) exhibit a plausible mass retrieval as deduced from the calculated refractive index increments d*n*/d*c* (excluding the contribution from non-proteinaceous material sedimenting slower than antibody monomers).

In contrast, the apparent mass retrieval of the undiluted antibodies (102–147 g/L) is substantially lower, unlike those measured at 25 krpm (see above). The dependency of retrieved signal from angular velocity is attributed to an artificially curtailed signal due to technical reasons (see above). This dependency is indicated by the different ordinate scales in the top panels of Fig. 12 showing experiments at 25 krpm and 40 krpm, respectively. This conclusion of an artificially reduced mass retrieval is in agreement with lacking evidence for any significant precipitation leading to a pronounced recovery loss. We refer to the limitations of steep boundary treatment as outlined above, if applied to measurements conducted at high angular velocities. These limitations apply for these results (40 krpm), as demonstrated by the excellent accordance when the very same sample is measured at 25 krpm. The J-O effect is manifest in about the same magnitude as described for the experiment at 25 krpm, as shown, for example, by the similar surplus of detected oligomers vs. dimers (Table 2).

In conclusion, the relative amounts of dimeric antibody and higher aggregates were overestimated by measurements at 40 krpm due to the artificially diminished retrieved signal.

### Concentration dependent analysis: Dilution series

To account for hydrodynamic non-ideality (co-exclusion and backflow effects), the sedimentation coefficients at each concentration were extrapolated to infinite dilution using the following equation [39–41].

$$s=s_{0}(1-k_{s}c)$$ (10)

As a reasonable assumption, the magnitude of all interactions, i.e. between antibody molecules of the same and different oligomeric state (self-concentration dependence and cross dependence), are considered to be equal as expressed by a single *k*_*s*_ constant. Equation 10 is a linear plot, frequently preferable for compact, near-spherical particles [41], whereas the reciprocal form of Equation 10 does not display a linear relationship of *1/s* vs. *c* over the whole concentration range of both antibodies.

This extrapolation allows for calculation of accurate molar masses of the main populations and thus their identification. Furthermore, it enables the quantification of the strength of unspecific intermolecular interactions via the interaction constant *k*_*s*_. The sedimentation coefficient at infinite dilution *s*_*0*_ is a matter constant under the specific solvent conditions.

**Fig 13:**
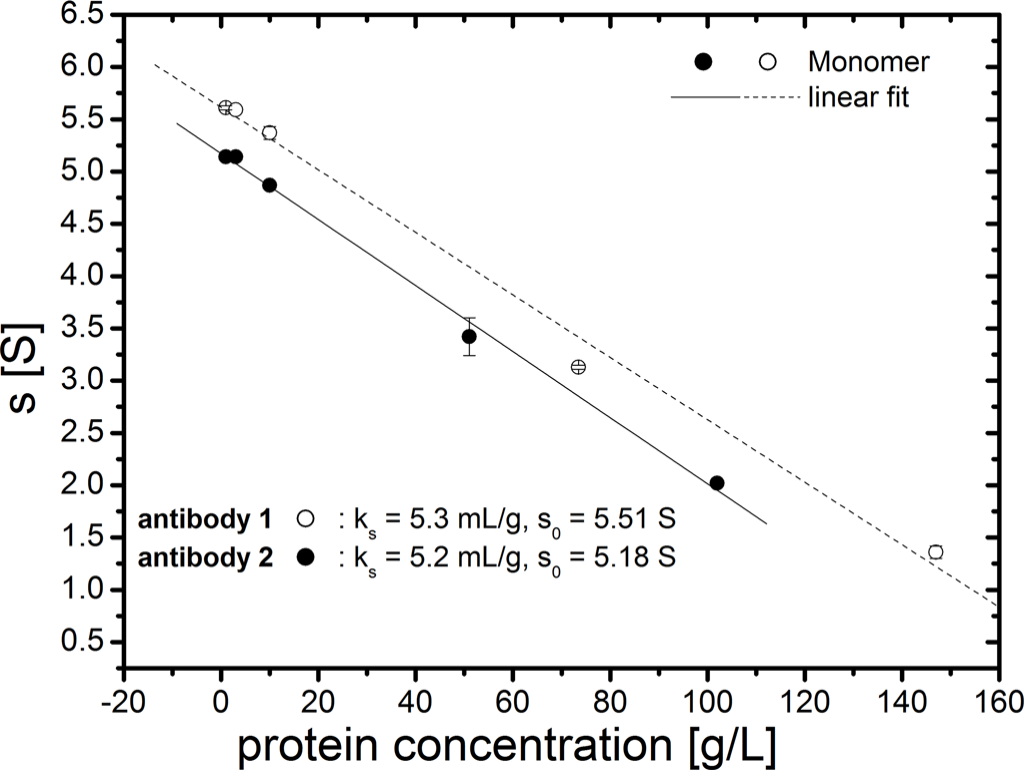
Plot of sedimentation coefficient vs. protein concentration.

Examples of the c(s) analysis for antibody 1 at 1–10 mg/mL are shown in S2 Fig. The ideal sedimentation coefficients *s*_*0*_ of the antibody monomers were determined to be 5.51 S and 5.18 S, respectively (Fig. 13), corresponding to molar masses of approx. 150 kDa calculated with a frictional ratio of 1.7. The interaction constant *k*_*s*_ for antibody monomers was found to be 5.3 mL/g and 5.2 mL/g, respectively (Fig. 10). These values for *k*_*s*_ are low, indicating weak intermolecular interactions. To put this result into perspective, uncharged globular proteins have the smallest contributions to non-ideality, in the order of 6 mL/g [17], while the theoretical minimum for proteins according to Rowe [40, 41] can be approximately assumed to be 3 mL/g, corresponding to a compact sphere (*f/f*_*0*_ = 1) with a partial specific volume typical for most proteins.

Additional valuable results were (1) the identification of non-proteinaceous populations by using protein-specific absorbance detection, (2) calculating mass balance, and (3) the monitoring of reversible/irreversible formation of oligomers/aggregates. Firstly, the non-proteinaceous nature of non-absorbing slowly sedimenting populations in both formulations is confirmed, since these were not detected by absorbance measurements using the XL-I ultracentrifuge. Secondly, as an indication for mass retrieval, the experimentally calculated refractive index increments and extinction coefficients are listed in S1 Table. The obtained values are typical for proteins. Accordingly, mass retrieval with both detection optics is plausible, confirming the results from undiluted antibody 1 measured at reduced rotational speed (see above) and thus, lack of evidence for irreversible aggregates in the two original formulations. Finally, the nearly quantitative recovery of antibody monomers at diluted conditions demonstrates the reversibility of the predominant proportion of oligomers in the original formulations. In conclusion, the results indicate reversible self-association in both formulations, with monomers clearly predominant at concentrations below 10 g/L.

## Discussion

The present sedimentation velocity analysis of highly concentrated proteins—aiming at an accurate oligomer distribution—expands the hitherto tractable protein concentration range. For the first time, the aggregation levels of highly concentrated antibodies were measured in their original formulations. Thus, any alterations in the size distribution which may arise due to substantial dilution and solvent change could be avoided. Indeed, size-exclusion chromatography (SEC) and other workhorse techniques used in pharmaceutical industries are prone to not accurately determine non-covalent aggregates, because of necessary dilutions and buffer changes [6–8].

The crucial technical challenge is the SV analysis of extremely steep, fast-moving boundaries, which are for the first time amenable to analysis using the unique Aida (Advanced Interference Detection Array) detector. Providing the technical premises for monitoring the concentration profiles of antibodies at concentrations up to at least 150 g/L in the present study is a major milestone in protein analytical chemistry. By developing a consistent experimental design and data fit approach based on the c(s) distribution method, we could achieve a robust quantification of soluble aggregates. Henceforth, the accurate quantification of soluble aggregates only awaits the comprehensive theoretical modelling of the non-ideal sedimentation behavior under those conditions.

If electrostatic interactions (primary charge effect) can be safely neglected as for antibodies in formulations with sufficient ionic strength, a comparatively low rotational speed along with floating *f/f*_*0*_ are the key measures to take into account non-ideal sedimentation behavior due to unspecific intermolecular interactions. Our results demonstrate that an insufficient diffusional spread of hypersharpened sedimentation boundaries, which is dependent on angular velocity, leads to an artificially curtailed total signal. In turn, the mass retrieval is incomplete and oligomer/aggregate levels are overestimated.

The present approach sacrifices diffusional information (as an obsolete frictional ratio) to account for steep sedimentation boundaries. In doing so, the amplitudes of the different peaks are treated as not affected by the pronounced non-ideal sedimentation behavior as a viable first-order approximation.

In fact, analyses that are based on the amplitudes of the found populations (representing the species populations for slowly interacting systems, such as self-associating antibodies) are much less susceptible to non-ideality-induced shifts in *s*-values as pointed out previously [13].

The validity of the results regarding the aggregate levels in the undiluted antibodies was supported by concentration-dependent analysis. The extent of non-ideal behavior was quantified by means of interaction constant *k*_*s*_ (assumed to be equal for all interactions between monomers and oligomers) and the identity of monomeric and dimeric antibodies could be verified. Calculating mass balance under dilute conditions showed that nearly all protein was recovered, of which 98–99% was in the monomeric state.

Most importantly, these findings demonstrate the near absence of insoluble aggregates/precipitates in the original highly concentrated formulations and the full reversibility of the predominant proportion of oligomers/aggregates. Nonetheless, the presence of soluble large oligomers/aggregates escaping detection cannot be excluded.

It should be noted that these measurements were only possible with the availability of samples with suitable properties: a highly concentrated antibody solution with comparatively low interparticle interactions (in terms of macroscopically visible low viscosity) allowing its sedimentation at all. In general, proteins with a higher diffusion coefficient than antibodies, e.g. globular proteins with lower masses than antibodies (< 150 kDa) should be amenable if interparticle interactions are low. However, experimental and data fit procedure may need to be optimized in specific cases. In the present case, the low magnitude of total concentration dependence results in a moderate J-O effect, usually increasing in all multicomponent systems with solute concentration, thus leading to an underestimation of antibody dimers and oligomers. In an extreme case of a pronounced J-O effect with convergence of the sedimentation coefficients of two components, a single hypersharp boundary is produced, obscuring the presence of two components, e.g. described for proteinpolysaccharides from bovine nasal cartilage at concentrations above 4 mg/mL [42]. In such a case, the amounts of the fast component appear to increase upon dilution.

The incorporation of the total effects of concentration-dependence into Lamm equation fitting has yet to be described in experimentally useful terms [43]. It may indeed turn out that the number of parameters required is larger than the precision of sedimentation velocity methodology can support [43].

Though the accuracy of the total aggregate content measured with the present method is uncertain due to the non-ideality effects which cannot be fully accounted for by available data fit procedures, it can be considered as a minimum value. Nonetheless, combined with the accurate aggregate quantification at low concentrations, crucial information for biopharmaceutical purposes is provided. This is particularly important, since no orthogonal methods are available as reference standards to evaluate the measured antibody aggregate content at such high concentrations.

The novel effects and challenges emerged will remain an interesting subject of fundamental research.

## Supporting Information

S1 Fig Example of c(S) analysis with different frictional ratios values applied for data fit of the same sedimentation velocity experiment. Data were aquired with the Aida interference detector at 25,000 rpm, 20°C. The upper top panels show raw data (circles) and best fit (lines). For clarity, only every fifth scan of the data set is shown. The respective c(s) distributions are shown in Fig. 5.
(TIF)

S2 Fig Example of c(S) analysis of diluted antibody 1 at 1–10~mg/mL. Sedimentation velocity experiments were conducted on an Optima XL-I ultracentrifuge at 40,000 rpm, 20°C. The upper top panels show raw data (circles) and best fit (lines). For clarity, only every fifth scan of the data set is shown. The lower top panels show best fit residuals of the plotted scans. The bottom panels show the c(s) distribution.
(TIF)

S1 Table Sedimentation velocity at 1–10 g/L measured with interference and absorbance (λ = 250 nm) optics. (ODS)
