# Supplementary figures and images for: Analysis of Antibody Aggregate Content at Extremely High Concentrations Using Sedimentation Velocity with a Novel Interference Optics
Source: PLoS One. 2015 Mar 24;10(3):e0120820. doi: 10.1371/journal.pone.0120820 (PMC4372433; doi:10.1371/journal.pone.0120820)

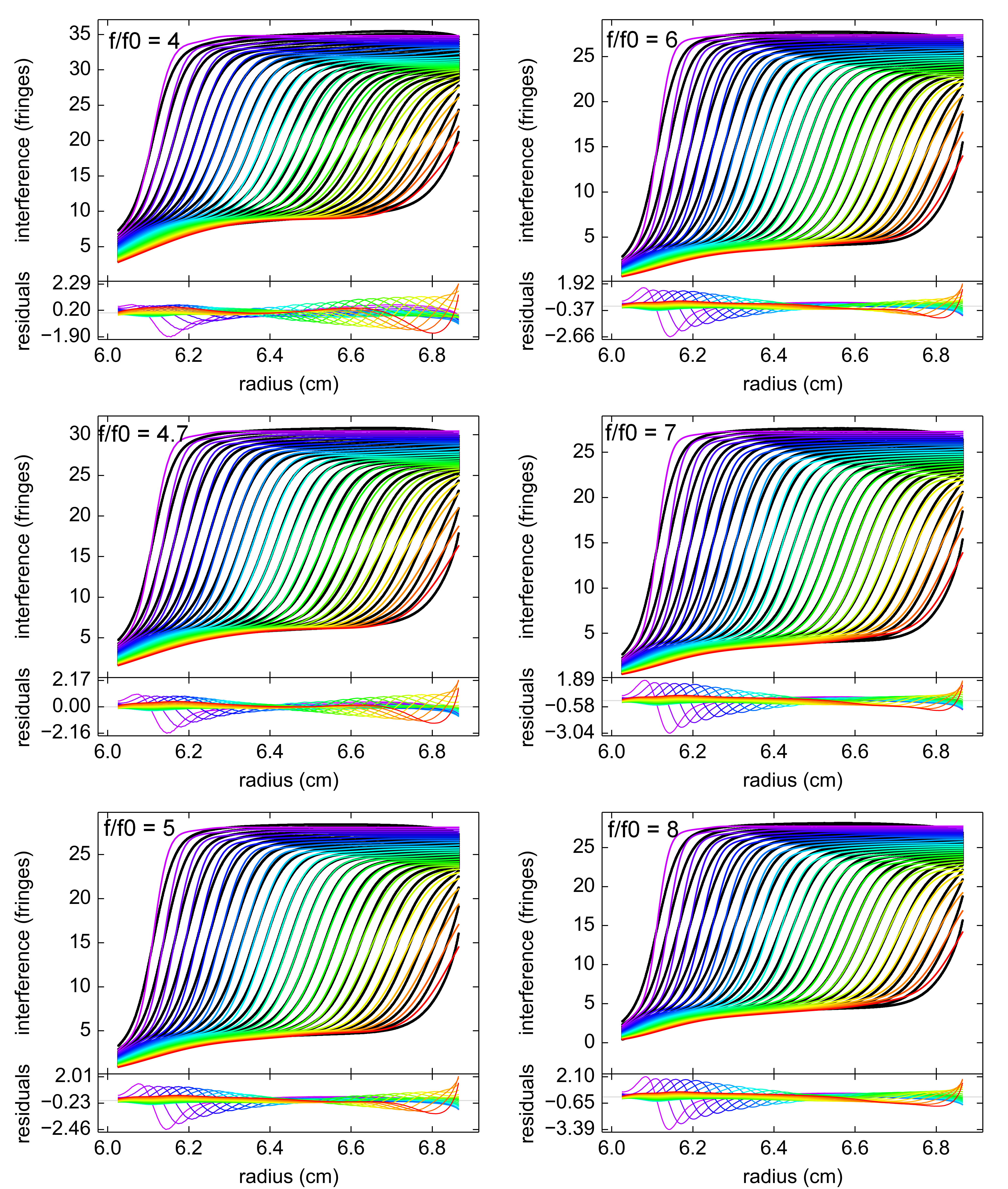

Supplement: S1 Fig — Data were aquired with the Aida interference detector at 25,000 rpm, 20°C. The upper top panels show raw data (circles) and best fit (lines). For clarity, only every fifth scan of the data set is shown. The respective c(s) distributions are shown in Fig. 5. (TIF) [file pone.0120820.s001.tif]

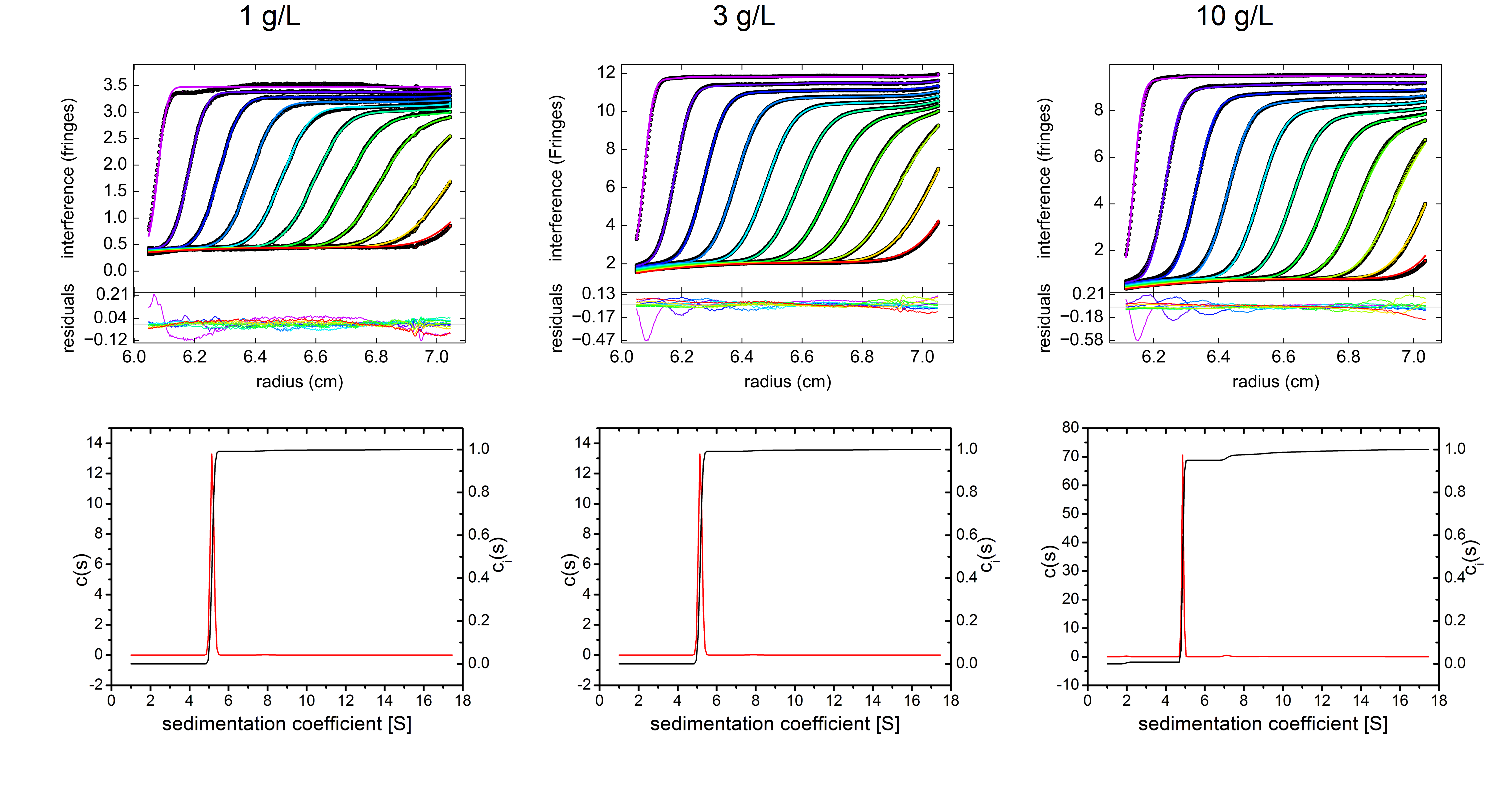

Supplement: S2 Fig — Sedimentation velocity experiments were conducted on an Optima XL-I ultracentrifuge at 40,000 rpm, 20°C. The upper top panels show raw data (circles) and best fit (lines). For clarity, only every fifth scan of the data set is shown. The lower top panels show best fit residuals of the plotted scans. The bottom panels show the c(s) distribution. (TIF) [file pone.0120820.s002.tif]
